# INTERPROFESSIONAL EDUCATION: STRATEGIES TO REACH RURAL STUDENTS THROUGH GERIATRIC EDUCATION

**DOI:** 10.1093/geroni/igad104.2116

**Published:** 2023-12-21

**Authors:** Elaine Jurkowski

**Affiliations:** Southern Illinois University at Carbondale, Cabondale, Illinois, United States

## Abstract

Inter-professional collaboration across disciplines such as primary care medical practice, physician assistants, social workers, and nutritionists does not occur naturally, since educational programs are often taught independently of each other, but these disciplines are required to work collaboratively with each other. The objective was to promote communication across disciplines and help each discipline understand the roles played in promoting mental health and general health for older adults. Three specific strategies to address Interprofessional Education were devised and implemented within a rural context to address the goal of improving collaboration and communication. An educational seminar was conducted using cases and guide questions focused on identifying strategies for care. A second strategy was through “Student hot-spotting” where students worked with a high-need complex case over the course of a school year weekly, as a team-based approach. A third strategy made use of a weekly medical clinic for team-based patient care. The teams consisted of Medical Residents, Social Work students, Physician Assistant students, and nutrition students. Findings suggest professionals were surprised at what they learned from the other disciplines they were collaborating with. They also learned about community-based resources available as well as strategies to promote health outcomes. All participants felt that the opportunity to collaborate outside of their disciplines would strengthen their impact when working with older adults and their families. In conclusion, a problem-based learning approach coupled with the opportunity to collaborate with other disciplines through (IPE) improves overall collaboration among professionals. All strategies had positive learning outcomes.

